# The genetic architecture of amylose biosynthesis in maize kernel

**DOI:** 10.1111/pbi.12821

**Published:** 2017-09-15

**Authors:** Changsheng Li, Yongcai Huang, Ruidong Huang, Yongrui Wu, Wenqin Wang

**Affiliations:** ^1^ College of Agriculture and Biology Shanghai Jiaotong University Shanghai China; ^2^ College of Agronomy Shenyang Agriculture University Shenyang China; ^3^ National Key Laboratory of Plant Molecular Genetics CAS Center for Excellence in Molecular Plant Sciences Institute of Plant Physiology & Ecology Shanghai Institutes for Biological Sciences Chinese Academy of Sciences Shanghai China; ^4^ University of the Chinese Academy of Sciences Beijing China

**Keywords:** maize, kernel, genome‐wide association study, amylose, starch, SNP

## Abstract

Starch is the most abundant storage carbohydrate in maize kernel. The content of amylose and amylopectin confers unique properties in food processing and industrial application. Thus, the resurgent interest has been switched to the study of individual amylose or amylopectin rather than total starch, whereas the enzymatic machinery for amylose synthesis remains elusive. We took advantage of the phenotype of amylose content and the genotype of 9,007,194 single nucleotide polymorphisms from 464 inbred maize lines. The genome‐wide association study identified 27 associated loci involving 39 candidate genes that were linked to amylose content including transcription factors, glycosyltransferases, glycosidases, as well as hydrolases. Except the *waxy* gene that encodes the granule‐bound starch synthase, the remaining candidate genes were located in the upstream pathway of amylose synthesis, while the downstream members were already known from prior studies. The linked candidate genes could be transferred to manipulate amylose content and thus add value to maize kernel in the breeding programme.

## Introduction

Maize is one of the most important crops in the world. Given the fact of ~10% protein and ~70% starch in the endosperm, it serves as a primary food for humans and feed for livestock (Nelson and Pan, 1995). There are mainly two forms of starch: the linear amylose and branched amylopectin. Amylose is composed of glucose residues linked through α‐1,4 bonds without any branch. Amylopectin contains both linear and branched glucose chains, 5% of which are joined via α‐1,6 bonds that introduce chain branches. The clusters of side chains allow amylopectin to fold into a dense and large molecule of glucose residues, which in turn increases kernel weight (James *et al*., 2003).

Starch in the normal maize endosperm is approximately 25% amylose and 75% amylopectin (Nelson and Pan, 1995). The ratio of amylose to amylopectin plays an important role in appearance, structure and quality of food product and processing. The amylose content determines the starch gelling and firmness, whereas the amylopectin is primarily responsible for the formation of crystalline granules and thickening of paste (Whitt *et al*., 2002). In general, high amylose improves the product texture of starch and turns it into a source of slowly digestible carbohydrate (Collins, 1909; Kim *et al*., 1998; Stinard *et al*., 1993), while low amylose corresponds to higher peak of paste viscosity and strong resistance to retrogradation (Van Hung *et al*., 2006).

**Figure 1:**
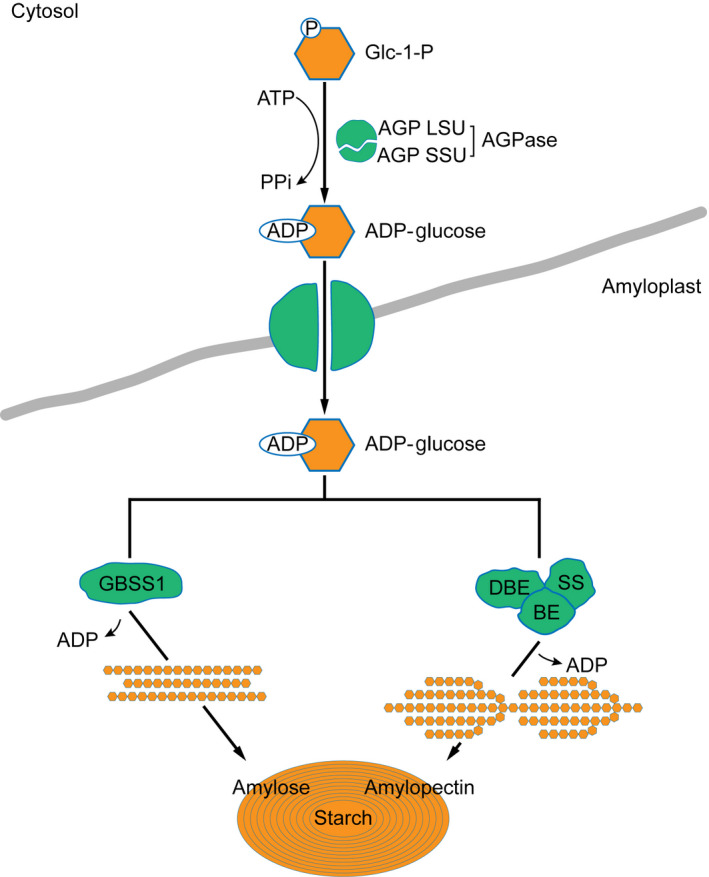
The elucidated pathway of starch biosynthesis in maize. The model summarizes the elucidated key enzymes involved in starch synthesis. Basically, ADP‐glucose as the glucosyl donor for starch biosynthesis is activated by ADP‐glucose pyrophosphorylase (AGPase), which is composed of two large subunits (AGP LSU) and two small subunits (AGP SSU). In amyloplast, the amylose is synthesized by granule‐bound starch synthase (GBSS), while amylopectin biosynthesis requires three more coordinated enzymes of soluble starch synthase (SS), starch‐branching enzyme (BE) and starch‐debranching enzyme (DBE).

Over the past decades, enormous progress has been made in understanding of the genetics and biochemistry of starch synthesis. The key enzymes involved in starch synthesis have been elucidated (Figure 1). Basically, the starch is synthesized by a suite of enzymes, including sucrose synthase (SUS), ADP‐glucose pyrophosphorylase (AGPase, the small unit encoded by *brittle2* (*bt2*) and the large by *shrunken2* (*sh2*)), soluble starch synthases (SSs) and granule‐bound starch synthase (GBSS, encoded by *waxy* (*wx*)), starch‐branching enzyme (BE, encoded by *amylose extender1* (*ae1*)) and starch‐debranching enzyme (DBE, encoded by *sugary1* (*su1*)) (Jeon *et al*., 2010). The starch synthesis pathway in maize endosperm is thought to begin with the cleavage of sucrose into fructose and UDP‐glucose, catalysed by SUS, the products of which are then converted into ADP‐glucose (ADPG) by AGPase. Amylose and amylopectin both use ADPG as the activated glucosyl donor for synthesis, but they are synthesized by different enzymes afterwards. GBSS is responsible for synthesis of amylose. Amylopectin biosynthesis requires a well‐coordinated machinery complex of enzymes including SSs, BE and DBE (Figure 1) (Hannah, 1997; James *et al*., 2003; Jeon *et al*., 2010; Keeling and Myers, 2010; Whitt *et al*., 2002). Lack of the BE enzyme in *ae1* leads to accumulation of up to 50% amylose due to the less amylopectin production. Mutations at the *waxy* locus eliminate amylose synthesis, resulting in 100% amylopectin in the endosperm. Both mutants have been used in the corn breeding to create either high‐ or low‐amylose maize to alter starch properties and utility. Reduction in amylopectin has been accomplished with *ae1* and *su1* mutants (James *et al*., 1995).

There is a dramatic variation in amylose content in the maize natural population ranging from zero (the waxy maize) to 64% (the amylomaize) (Mercier, 1973). Elucidating the genetic variation in starch biosynthesis and regulation is laborious based on traditional QTL mapping. Genome‐wide association study (GWAS) has become a promising way to decipher the causal relationship between genetic polymorphisms and biological traits in plants. The maize inbred lines via self‐cross are ideal materials to conduct GWAS. A number of agronomic traits in maize such as mercury accumulation (Zhao *et al*., 2017), starch content (Liu *et al*., 2016b), drought tolerance (Wang *et al*., 2016) have been successfully analysed. The GWAS identified 37 and 74 loci significantly associated with mercury accumulation and kernel oil concentration in maize, respectively (Li *et al*., 2013; Zhao *et al*., 2017). The recent GWAS for total starch content in maize kernel identified four significant SNPs and 77 candidate genes including a key gene of glucose‐1‐phosphate adenylyltransferase (Liu *et al*., 2016b). Still, the complete network and regulation involved in specific amylose or amylopectin biosynthesis is limited. Here, we are aiming to detect SNPs that are significantly associated with amylose accumulation, which may aid in enhancing the real value of starch in maize kernel. The further functionally validated candidate genes can be readily transferred to maize and other species using genetic engineering biotechnology.

## Results

### Natural variation in the amylose content

**Figure 2:**
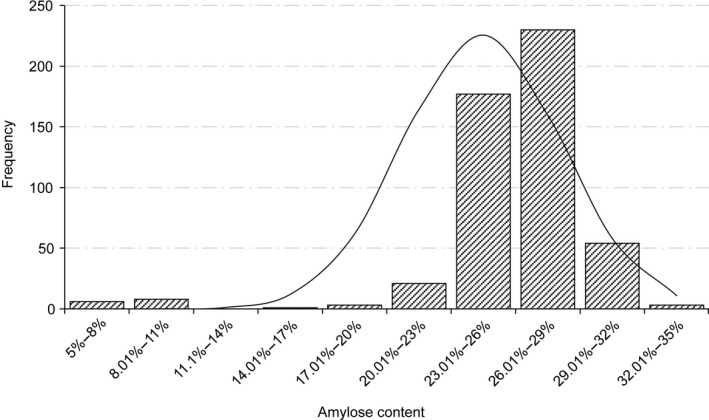
The frequency distribution of amylose content. *x*‐axis shows the amylose content, and *y*‐axis shows the population frequency.

The amylose/amylopectin composition in starch is a complex quantitative trait affecting the quality and yield of maize endosperm. In order to improve the seed quality by manipulating target genes, we selected a mapping population of 464 lines from a natural‐variation germplasm pool with a wide genetic diversity. There was no obvious division of subgroups in our population, which could eliminate false positive and improve the power to recover meaningful associations. We measured the amylose content for the 464 maize lines including 454 natural inbred lines and 10 *waxy* lines that contain low amylose (Table S1) using a modified iodine colorimetry. There was a great variation in the amylose content ranging from 7.38% to 32.82% in the association panel. The overall distribution exhibited left‐skewed due to the presence of *waxy* maize materials (Figure 2). The average kernel amylose content was 25.93%, close to 25% in the normal maize seed.

### Linkage disequilibrium

**Figure 3:**
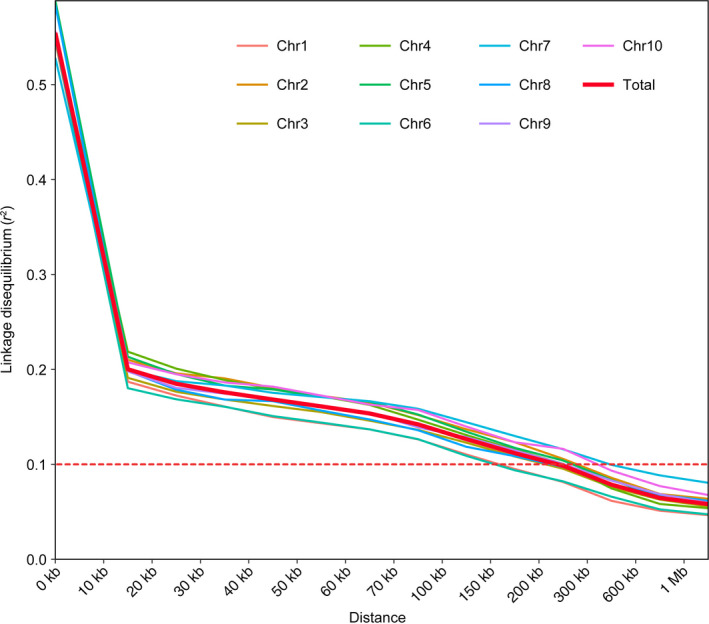
Linkage disequilibrium decay on maize chromosomes and whole genome. The red dashed horizontal line shows the LD threshold for the association panel (*r* ^2^ = 0.1).

Developed by Lai's group (Liu *et al*., 2016a), 9 007 194 single nucleotide polymorphisms (SNPs) with a minor allele frequency (MAF) more than 0.05 could cover the whole maize genome and are theoretically sufficient for efficient GWAS analysis in maize with the genome size of 2 300 MB. We took advantage of these qualified SNPs as input data to calculate the genome‐wide linkage disequilibrium (LD) in the association panel. We found a rapid decline in LD with the increasing physical distance on all chromosomes. The mean length of LD decay decreased rapidly to 15 kb at a cut‐off of *r* ^2^ = 0.2 (Figure 3). The decay rate varied among chromosomes with a shortest LD (150 kb, *r* ^2^ = 0.1) estimate observed on chromosome 6 and the longest LD (450 kb, *r* ^2^ = 0.1) observed on chromosome 7. The overall LD decay distance was 250 kb (*r* ^2^ = 0.1) across the entire genome (Figure 3).

### Loci associated with the amylose content

**Figure 4:**
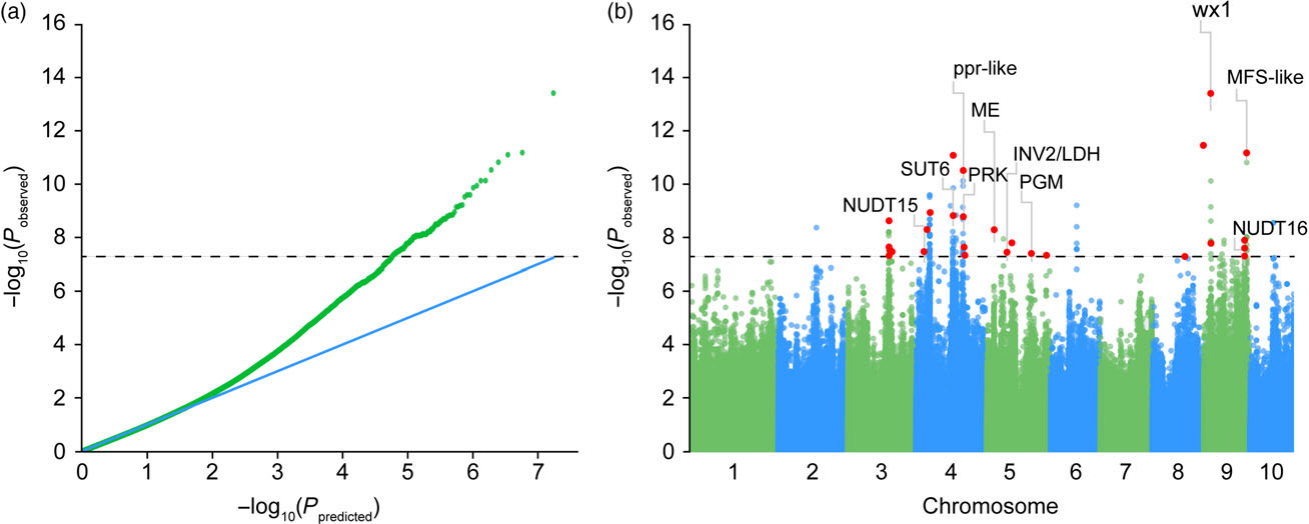
Quantile–quantile and Manhattan plots for the association study of amylose content in maize kernels. (a) Quantile–quantile for amylose content. (b) Manhattan plot of amylose content. The dashed line indicates the significance threshold of *P*‐value 5 × 10–8. 27 unique SNPs are labelled with red dots, and the corresponding genes in the amylose/amylopectin of carbohydrate metabolic pathway are highlighted.

**Figure 5:**
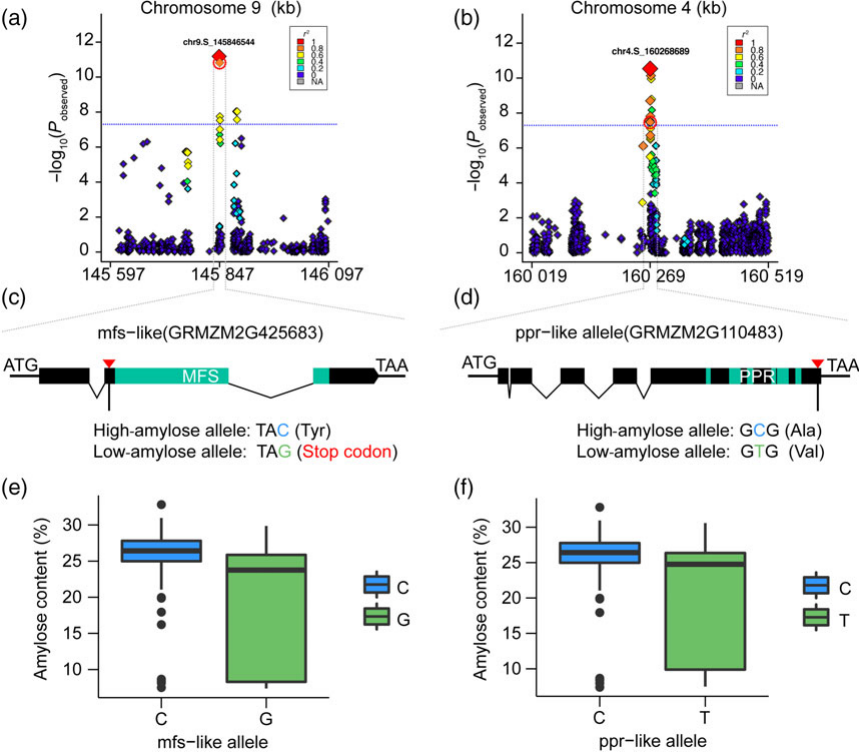
Candidate causative genes and variants underlying amylose content in maize kernel. (a) and (b) Regional Manhattan plot of the mfs‐like genomic region on chromosome 9 and the ppr‐like genomic region on chromosome 4. The 250‐kb genomic region on either side of the most significant SNP is shown. The lead SNP is shown with a largest red diamond. The red broken circle indicates the SNPs in coding region for the corresponding genes. (c) and (d) Candidate causative variants in mfs‐like and ppr‐like. Red nabla shows the locations of SNPs. The green colour showed the predicted coding region. (e) and (f) Allele effects for corresponding SNPs in mfs‐like and ppr‐like genes.

GWAS was performed to associate the phenotype of amylose content with the genotype of 464 diverse inbred lines. Given the population structure and familial relatedness in the natural population, we applied the mixed linear model (MLM) to decrease the false‐positive error. Quantile–quantile plots (Figure 4a) showed that the observed distribution did not deviate from the expected line, indicating that the confounders did not have significant influence. However, the curved tail represented a small number of true associations with the amylose content among thousands of unassociated SNPs. Association analysis identified 42 SNPs significantly associated with the amylose content at the minimum of *P *≤* *5 × 10^−8^ (Figure 4b and Table S2). Due to the overall LD decay distance being 250 kb (*r* ^2^ = 0.1) across the entire genome (Figure 3), a 250‐kb region flanking the left and right sides of each SNP was defined as a QTL. 352 genes identified within the defined QTLs are summarized in Table S2. Of them, 39 candidate genes at 27 QTLs were implicated in carbohydrate metabolism and regulation (Table 1). The most significant SNP (chr9.S_23283117) was linked with *waxy1* (~22 kb away of GRMZM2G024993) (Figure 4b), a key gene encoding GBSS for amylose biosynthesis (Shure *et al*., 1983). Four SNPs exist in gene regions, namely two (chr9.S_145846544) in the locus of GRMZM2G425683 (Figure 5a), one (chr4.S_160268689) located in GRMZM2G110483 (Figure 5b) and one in AC204428.4_FGT004 with unknown function yet. GRMZM2G425683 encodes a major facilitator superfamily protein (MFS‐like) responsible for sugar transport. The two SNPs in GRMZM2G425683 create a stop codon and a nonsynonymous amino acid substitution (Figure 5c). The allele of C was greatly correlated with a high amylose content, while the allele of G was linked with a low level of amylose (Figure 5e). Surprisingly, GRMZM2G425683 was specifically expressed in roots, but not in the seeds (Stelpflug *et al*., 2016). GRMZM2G110483 encodes a pentatricopeptide repeat‐containing protein (PPR‐like), which is highly expressed in the endosperm. The SNP in the PPR‐like gene causes a C‐to‐T mutation leading to an Ala‐to‐Val amino acid substitution (Figure 5d). The allele for high amylose is C compared with the T allele for low amylose (Figure 5f). Still, further investigation is required to test whether GRMZM2G425683 and GRMZM2G110483 affect the amylose content.

**Table 1:** SNPs and candidate genes significantly associated with amylose content

SNP ID	Allele	*P* value	Associate genes	Annotation	Symbol
chr3.S_139436833	C/T	2.06E−08	GRMZM2G174769	Putative galacturonosyltransferase‐like 9	GAUT‐like9
chr3.S_139499410	A/G	2.22E−09	GRMZM2G340756	MYB‐related‐transcription factor	MYBR14
chr3.S_140663351	A/G	4.61E−08	GRMZM2G402368	Alpha‐galactosidase 3	GLA3
chr3.S_141284190	C/T	2.77E−08	GRMZM2G073584	Beta galactosidase 9	lacZ9
chr3.S_141284190	C/T	2.77E−08	GRMZM2G067171	Putative GATA transcription factor	GATA31
chr3.S_149274688	A/T	3.12E−08	GRMZM2G127789	Glutathione S‐transferase GST 29	GST29
chr4.S_125184357	A/G	1.43E−09	GRMZM2G106741	Sucrose transporter 6	SUT6
chr4.S_125294997	G/T	7.84E−12	GRMZM2G312806	Mitochondrial transcription termination factor	mTERF
chr4.S_159118307	A/G	1.57E−09	GRMZM2G026024	Phosphoribulokinase	PRK
chr4.S_160268689	C/T	2.89E−11	GRMZM2G110483	Pentatricopeptide repeat‐containing protein	PPR‐like
chr4.S_160268689	C/T	2.89E−11	GRMZM2G589696	DOF‐transcription factor 43	dof43
chr4.S_160268689	C/T	2.89E−11	GRMZM2G143804	Phosphoribulokinase	PRK
chr4.S_163058675	A/G	2.19E−08	AC186147.3_FG008	Alpha‐6‐galactosyltransferase	x34.3
chr4.S_165621095	C/T	4.31E−08	GRMZM2G122846	bZIP transcription factor	bZIP
chr4.S_25408622	C/G	3.08E−08	GRMZM5G809417	Nudix hydrolase 15	NUDT15
chr4.S_37345698	A/G	4.76E−09	GRMZM2G008482	Orphans transcription factor	Orphans
chr4.S_37345698	A/G	4.76E−09	GRMZM2G363540	Glutathione S‐transferase GST 26	GST26
chr4.S_46702131	C/G	1.09E−09	GRMZM2G100583	NAC domain transcription factor	NAC75
chr4.S_46744218	C/T	2.49E−10	GRMZM2G129090	UDP‐glycosyltransferase 91D1	UGT91D1
chr5.S_149999559	C/T	3.71E−08	GRMZM2G173674	Phosphoglucomutase	PGM
chr5.S_202171324	A/C	4.45E−08	GRMZM2G164912	Galactan beta‐1,4‐galactosyltransferase	GALS1
chr5.S_24096526	A/G	4.71E−09	GRMZM2G085747	Malic enzyme	ME
chr5.S_67474748	A/C	3.28E−08	GRMZM2G128929	L‐lactate dehydrogenase	LDH
chr5.S_67474748	A/C	3.28E−08	GRMZM2G089836	Invertase 2	INV2
chr5.S_83843824	A/G	1.48E−08	GRMZM2G449843	Alpha/beta‐Hydrolases	ABHD
chr8.S_110374862	C/T	4.87E−08	GRMZM2G039017	3‐beta‐glycosyltransferase	B3GALT
chr9.S_138336082	C/T	4.52E−08	GRMZM2G118979	Alpha/beta‐Hydrolases	ABHD
chr9.S_138363042	C/G	2.40E−08	GRMZM2G095727	Two‐component response regulator‐like PRR73	ARR‐B‐like
chr9.S_139318861	A/C	1.20E−08	GRMZM2G165357	UDP‐glucuronic acid decarboxylase 1 isoform	UXS1
chr9.S_139318861	A/C	1.20E−08	GRMZM2G161293	Beta‐1‐3‐galactosyl‐O‐glycosyl‐glycoprotein	BGGP
chr9.S_145846544	C/T	6.52E−12	GRMZM2G356579	Calmodulin‐binding transcription activator 2‐like	CAMTA2
chr9.S_145846544	C/T	6.52E−12	GRMZM2G026742	HSF‐transcription factor 9	hsftf9
chr9.S_145846544	C/T	6.52E−12	GRMZM2G126936	NAC domain‐containing protein 67‐like	nactf45
chr9.S_145846544	C/T	6.52E−12	GRMZM2G425683	Major facilitator superfamily protein	MFS‐like
chr9.S_145846544	C/T	6.52E−12	GRMZM2G126834	ARR transcription factor	arr1
chr9.S_23283117	C/T	3.87E−14	GRMZM2G024993	Granule‐bound starch synthase	GBSS
chr9.S_23283117	C/T	3.87E−14	GRMZM2G171395	NAC domain transcription factor	nactf86
chr9.S_24270258	A/G	1.60E−08	GRMZM5G811192	Glycosidases	GH81
chr9.S_24270258	A/G	1.60E−08	GRMZM2G069008	Nudix hydrolase 14	NUDT

Association analysis identified 27 SNPs and 39 candidate genes implicated in carbohydrate metabolism and regulation.

### Functional annotation for candidate genes

Besides the known *waxy* gene involved in amylose synthesis, we identified more genes such as GRMZM2G089836, GRMZM2G173674 and GRMZM2G069008 from the upstream of the starch synthesis pathway. For example, GRMZM2G089836 encodes an invertase. It was reported that sucrose entering the seed has to be cleaved by invertase into glucose and fructose, which are then resynthesized into sucrose (Walley *et al*., 2016; Wind *et al*., 2010). The mutant of the *Arabidopsis* cytosolic invertase had a high sucrose‐to‐glucose ratio and grew shorter primary roots (Qi *et al*., 2007). The maize *miniature1* (*mn1*), a mutant with lack of cell wall invertase2, reduced the sucrose transport to the seeds, which resulted in 30% loss of seed weight (Kang *et al*., 2009). The gene of GRMZM2G173674 encodes a phosphoglucomutase (PGM). It was studied in potato that phosphoglucomutase catalysed the conversion of glucose‐6‐phosphate into glucose‐1‐phosphate that acted as a precursor for starch biosynthesis in plastids (Van Harsselaar *et al*., 2017). The gene was broadly expressed in all tissues, especially in immature cob and early stage of seed development (Walley *et al*., 2016). It was found that the plastidic phosphoglucomutase expression was up‐regulated when starch began to accumulate, while its mutant impaired starch synthesis in rice pollen grains and caused male sterility (Lee *et al*., 2016). GRMZM2G069008 encodes a Nudix hydrolase that is widely distributed in dicots and monocots. Its main function catalyses the cleavage of ADP‐glucose linked to starch biosynthesis (Kraszewska, 2008). It was reported that the pattern of ADP‐glucose hydrolytic activity was inversely correlated with starch accumulation (Rodriguez‐Lopez *et al*., 2000). One of Nudix hydrolases reduced the levels of both ADP‐glucose and the amylose content when it was overexpressed in *Arabidopsis* (Ge and Xia, 2008; Munoz *et al*., 2006). Its decreased expression at the middle stage of maize endosperm development was consistent with the amylose content (Chen *et al*., 2014).

The 39 potential genes at 27 associated loci were annotated as nine transcription factors, four glycosyltransferases, three glycosidases and four hydrolases, as well as 19 other members (Table 1). The transcription factors included MYB, GATA, bZIP, DOF, HSF, NAC, ARR superfamily, indicating their multiple functions and regulations in amylose metabolism (Table 1). Glycosyltransferases and glycosidases form the major catalytic machinery for the synthesis and breakage of glycosidic bonds in disaccharides, oligosaccharides and polysaccharides (Hancock *et al*., 2006; Lairson *et al*., 2008; Liang *et al*., 2015). The enriched glycosyltransferases and glycosidases from the GWAS analysis indicated their important role in amylose metabolism.

**Figure 6:**
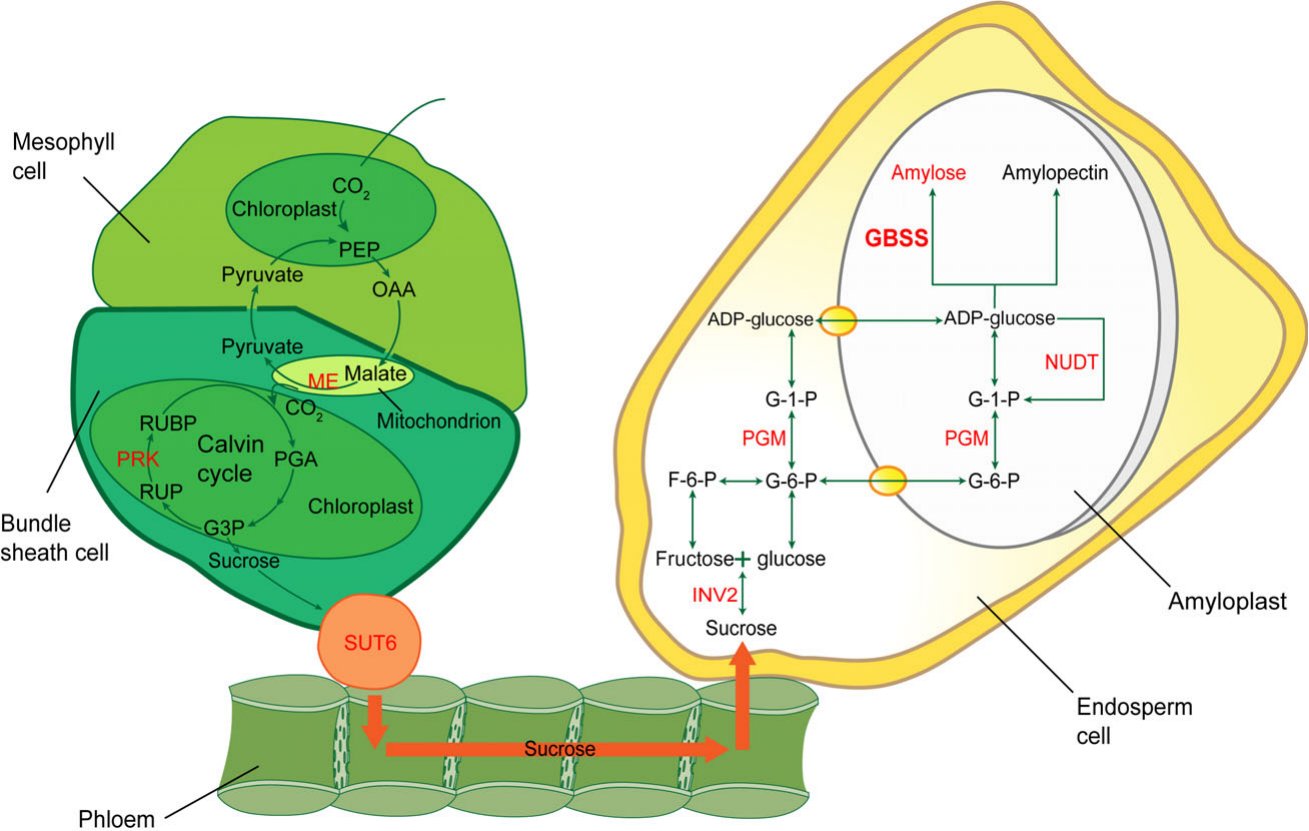
A model of amylose/amylopectin biosynthesis pathway. The model is summarized based on the previous knowledge and our GWAS analysis. In leaves, sucrose is produced from the Calvin cycle by carbon fixation with the activity of mitochondrial NAD‐dependent malic enzyme (ME encoded by GRMZM2G085747), phosphoribulokinase (PRK encoded by GRMZM2G026024 and GRMZM2G143804) and other enzymes. Then, sucrose is transported through phloem to the storage organ of endosperm by sucrose transporter (SUT6 encoded by GRMZM2G106741), where it is imported into the cytosolic compartment of each cell. In the cytosol, the sucrose synthase (SUS) or invertase (INV encoded by GRMZM2G089836) cleaves sucrose into fructose and UDP‐glucose. After glucose 6‐phosphate is transported into amyloplasts, phosphoglucomutase (PGM encoded by GRMZM2G173674) catalyses glucose 6‐phosphate into glucose 1‐phosphate that is activated into ADP‐glucose as the substrate for starch synthesis. The content of ADP‐glucose is also negatively regulated by Nudix hydrolases (NUDT encoded by GRMZM5G809417 and GRMZM2G069008) that break down ADP‐glucose linked to starch biosynthesis. Granule‐bound starch synthase (GBSS encoded by GRMZM2G024993) cooperates with other enzymes to catalyse and elongate sugar chains. All enzymes encoded by candidate genes found from GWAS are highlighted in red colour.

In conclusion, we propose to integrate the previous knowledge with our GWAS analysis into a model pathway of amylose biosynthesis in maize endosperm (Comparot‐Moss and Denyer, 2009; Keeling and Myers, 2010; Zeeman *et al*., 2010). Sucrose is produced from the carbon fixation of Calvin cycle in leaves through the activity of mitochondrial NAD‐dependent malic enzyme (ME encoded by GRMZM2G085747), phosphoribulokinase (PRK encoded by GRMZM2G026024 and GRMZM2G143804) and other enzymes. Then, sucrose is transported to the storage organ by sucrose transporter (SUT6 encoded by GRMZM2G106741), where it is imported into the cytosolic compartment of each cell. In the cytosol, the sucrose synthase (SUS) or invertase (INV encoded by GRMZM2G089836) cleaves sucrose into fructose and UDP‐glucose. After glucose 6‐phosphate is transported into plastids, phosphoglucomutase (PGM encoded by GRMZM2G173674) catalyses glucose 6‐phosphate into glucose 1‐phosphate that is activated into ADP‐glucose as the substrate for amylose synthesis. The content of ADP‐glucose is also negatively regulated by Nudix hydrolases (NUDT encoded by GRMZM5G809417 and GRMZM2G069008). GBSS encoded by GRMZM2G024993 cooperates with other glycosyltransferase (AC186147.3_FG008, GRMZM2G129090, GRMZM2G164912, GRMZM2G039017) and glycosidase (GRMZM2G402368, GRMZM2G073584, GRMZM5G811192) to catalyse and elongate the sugar chains (Figure 6).

## Discussion

It is well known that after the key enzyme of AGPase catalyses 1‐P‐glucose into the activated form of ADP‐glucose for amylose and amylopectin biosynthesis, the downstream enzymes determine their destinies. Amylose is synthesized by GBSS, whereas amylopectin production is coordinately catalysed by SSs, BE and DBE (Figure 1). It is not surprising that a prominent association peak in the QTL region of the *waxy* locus (GRMZM2G024993) on chromosome 9 was highly linked with the amylose content. The other QTLs might contribute to both amylose and amylopectin biosynthesis (Figure 6). The w*axy* gene encoding GBSS plays a critical role in amylose synthesis (Comparot‐Moss and Denyer, 2009; Jeon *et al*., 2010; Stamp *et al*., 2016). The dynamic transcriptome analysis showed that *waxy* was actively expressed in the middle phase of maize endosperm development (Chen *et al*., 2014). Mutations in *waxy* eliminate amylose but increase the amylopectin content during the grain filling. These *waxy* mutants have been used in breeding to create high‐amylopectin maize (Stamp *et al*., 2016; Tsai, 1974). Recent studies were focused on the downstream genes involved in the final product of starch biosynthesis, such as *bt2*,* sh2*,* su2*,* ae1*,* su1* (Figure 1). Due to the complex of starch biosynthesis and small effect from multiple QTLs, the genetic mechanism of amylose itself rather than total starch synthesis is challengeable to investigate. Taking advantage of GWAS analysis, we were able to detect almost all QTLs at a genome‐wide level with sufficient accuracy and sensitivity. Noticeably, the enzymes in the upstream of amylose pathway were deciphered, such as sucrose transporter, invertase, phosphoglucomutase, Nudix hydrolase, glycosyltransferases and glycosidases that are responsible for the precursors for amylose biosynthesis (Figure 6).

The 2,300 Mb of maize genome includes ~32,000 genes with large intergenic regions and ~84% repeats (Jiao *et al*., 2017). On average, every 72 kb contains a gene. Thus, it is reasonable that we found *waxy* being 22 kb away from one of the most significant SNPs with a *P*‐value of 3.86e−14. The acceptable distance between SNPs and candidate genes relies on species, genotype structure and the linkage disequilibrium decay distance. For example, the GWAS for seed oil melting point in *Arabidopsis* identified two candidate genes *FAD2* (AT3G12120) and *FATB* (AT1G08510) being 74 and 55 kb away from their corresponding SNPs. Molecular studies of FAD2 and FATB variants confirmed that they influence the quantities of the fatty acids (Branham *et al*., 2016). In the GWAS analysis of oil biosynthesis in maize, the distance from the lead SNP was applied as 50 kb to define potential candidate genes (Li *et al*., 2013). The 200‐kb genomic region on either side of the most significant SNP is determined as acceptable distance for the genome‐wide study of drought tolerance in maize (Wang *et al*., 2016).

GWAS has begun to serve as a new foundation for understanding the genetic architecture of complex agronomic traits in crops, although it still has confounding limitations. GWAS is powerful to analyse traits underpinned by a small number of loci with large effect size. It should be noticed that the identified significant 42 SNPs are not always the true causative loci by the facts of linkage or error structure of data. We need to take into account of sample size, the imperfect genotyping data and other confounding factors. We maximized the genetic variance within 464 samples collected from different locations. We only used inbred lines to keep genetically identical, which are suited to GWAS analysis. We found 39 candidate genes from 27 significant loci associated with the amylose content. Surprisingly, we did not find any overlapped genes such as *sh2* and *bt2* in the known pathway of starch biosynthesis. The reason could be due to small effect of multiple QTLs contributing to the trait of amylose/amylopectin content. Additionally, rare variants suffer from many other noncausative rare variants with strong or complete association within the genome (Korte and Farlow, 2013).

## Experimental procedures

### Plant material

The GWAS mapping population included 464 temperate maize inbred lines collected and genotyped by Dr. Lai's laboratory at China Agricultural University (Liu *et al*., 2016a). All the inbred lines were planted in the field of Sanya (Hainan Province, 18.75N, 109.17E) in November 2013. The mature seeds of each inbred line were harvested in bulk and used for amylose content analysis.

### Amylose content determination

Apparent amylose content (AAC) was measured based on the modified protocol from Gibbon *et al*. (Gibbon *et al*., 2003). Mature kernels were ground lightly in a grinding mill. Instead of total starch isolation, we filtered the kernel flour with 80‐mesh nylon screen and dried the flour at 37 °C in oven overnight. We weighed 25 mg kernel flour into 15‐mL centrifuge tube, added 4 mL 80% ethanol and mixed well, heated in a water bath at 80 °C for 20 min with a mixing every 5 min, then centrifuged at 1500 g for 10 min, and supernatant was poured. The precipitates were gelled in 300 μL of absolute ethyl alcohol and 4.5 mL of 1M NaOH by heating to 65 °C for 1 h; subsequently, 500 μL sample was transferred to 50‐mL centrifuge tube, diluted by adding 25 mL water and neutralized in 1 mL 1 m acetic acid, then added 1 mL I_2_/KI solution (2 mg/mL I_2_/20 mg/mL KI) and made constant volume to 50 mL, mixed well and incubated for 10 min. The OD was measured on a spectrophotometer (Beckman Coulter) using a 620‐nm filter. The amylose content was determined by the average value from three biological replicates.

### Association mapping and SNP annotation

The genotypes of 9,007,194 SNP markers filtered to retain only MAF >0.05 were generated by Dr. Lai's laboratory at China Agricultural University (Liu *et al*., 2016a). The decay distance of linkage disequilibrium in the association panel was calculated by PopLDdecay (https://github.com/BGI-shenzhen/PopLDdecay) with all SNP markers and mixed linear model (MLM) GWAS was carried out using the EMMAX suite (Kang *et al*., 2010). EMMAX‐kin was used to calculate the Balding–Nichols kinship matrix to account for family relatedness among the samples, after which EMMAX associations with the phenotype were calculated. The significant threshold of *P* value is 5e−8, indicated by a horizontal line in the Manhattan plot at −log_10_ (*P* value) = 7.3.

All SNPs significantly related to the phenotype were annotated by snpEff (Cingolani *et al*., 2012). The SNP with the most significance within the same LD block (*r* ^2^ < 0.1) was selected to represent the locus. The LD between SNPs was also calculated by PopLDdecay (https://github.com/BGI-shenzhen/PopLDdecay). To seek candidate genes in the identified loci for the amylose, we used public gene annotation data sets from both maizeGDB (http://maizegdb.org) and NCBI (https://www.ncbi.nlm.nih.gov). All the annotated genes within the decay distance of linkage disequilibrium in the association panel of SNPs were retrieved. Blastp, InterProScan (Jones *et al*., 2014) and Pfam (Finn *et al*., 2014) were also used for annotation candidate genes.

## Conflict of interest

The authors have no conflict of interest to declare.

## Supporting information


**Table S1** Amylose content for 464 maize lines.

**Table S2** Significantly associated SNPs identified by GWAS in maize.
